# Clinical Laboratory Stressors Used to Study Alcohol–Stress Relationships

**DOI:** 10.35946/arcr.v34.4.10

**Published:** 2012

**Authors:** Suzanne Thomas, Amy K. Bacon, Rajita Sinha, Magdalena Uhart, Bryon Adinoff

**Affiliations:** **Suzanne Thomas, Ph.D.,***is associate professor of psychiatry at the Medical University of South Carolina, Charleston, South Carolina.*; **Amy K. Bacon, Ph.D.,***is assistant professor of psychology at Bradley University, Peoria, Illinois.*; **Rajita Sinha, Ph.D.,***is professor of psychiatry at Yale University School of Medicine, New Haven, Connecticut.*; **Magdalena Uhart, M.D.,***is assistant professor of medicine in the Department of Medicine at The Johns Hopkins University School of Medicine, Baltimore, Maryland, and assistant professor of medicine, Department of Medicine, Cedars-Sinai Medical Center, David Geffen School of Medicine at the University of California, Los Angeles, California.*; **Bryon Adinoff, M.D.,***is a Distinguished Professor in the Department of Psychiatry, University of Texas Southwestern Medical Center, Dallas, Texas, and staff psychiatrist at the Veterans Affairs North Texas Health Care System, Dallas, Texas.*

**Keywords:** Alcohol consumption, alcohol use and abuse, alcoholism, stress, stressor, physiological stressor, psychological stressor, pharmacological stressor, biological adaptation to stress, stress reactivity, stress-induction study, clinical study, laboratory study, controlled study

## Abstract

Understanding the biologic systems that underlie the relationship between stress and alcohol consumption may lead to better prevention efforts and more effective treatments for alcoholism. Clinical laboratory studies offer a unique opportunity to examine these relationships by using a controlled environment to study how an acute stressor affects alcohol drinking and alcohol craving, how individuals in recovery or those at risk for alcoholism may respond differently to stressors relative to control subjects, and how alcohol differentially affects stress reactivity in these groups. This article reviews some of the most common physical, psychological, and pharmacological stressors used in stress-induction studies designed to reveal details about the relationship between stress reactivity and alcohol use and abuse.

A comprehensive understanding of the relationship between stress and alcohol use is important for understanding the risks of developing alcohol problems and subsequent relapse. Although the relationship is complex, substantial evidence supports that exposure to chronic stress early in life (e.g., Sher et al. 1997), adult trauma (Kessler et al. 1995), and the presence of anxiety disorders (Grant et al. 2004) all are associated with increased prevalence of alcohol use and risk of developing of an alcohol use disorder. Although people with high levels of stress may report that they use alcohol to reduce stress (Thomas et al. 2003), there is inconsistent evidence that stress promotes subsequent drinking (Helzer et al. 2006; Park et al. 2004; Todd et al. 2009). Likewise, inconsistent evidence exists as to whether inducing stress in people with alcohol dependence leads to craving or drinking (Cooney et al.1997; Fox et al. 2007; Ray 2011; Thomas et al. 2011*a*,*b*) or whether alcohol use actually relieves stress (see Sayette 1999). Even so, stress is a frequently cited reason for relapse by people with alcohol dependence, and most evidence-based treatments for alcohol dependence include stress coping and mood management (Marlatt and Gordon 1985; Vieten et al. 2010).

The complexity of this issue warrants investigation with well-controlled studies. With clinical laboratory studies, researchers can conduct experiments to establish causal relationships between stress and alcohol use. In contrast to studying stress and drinking in the real world, the clinical laboratory setting allows scientists to carefully calibrate and apply a stressor, to administer different types of stressors, and to assess the interaction among multiple pre-existing variables (e.g., genotype, temperament, drinking motives, alcohol expectancies, or comorbid psychiatric conditions) and stress response variables (i.e., subjective, physiological, and neuroendocrine responses). Such studies permit the study of sensitivity or resilience to acute stressors in at-risk or currently dependent individuals, of how alcohol can differentially reduce stress reactivity in different groups of participants, or how and whether a stressor induces alcohol craving or consumption.

## A Review of Clinical Laboratory Stressors

This article reviews some of the most common methods used to induce a stress response in participants in a clinical laboratory setting. The stressors are divided into three main categories: physical, psychological, or pharmacologic. As explained throughout this article, the best stressor to use depends on the research question of interest.

## Physical Stressors

Physical stressors, such as pain, exercise, or extreme temperatures, are communicated directly to the hypothalamus by way of the nervous system (Herman and Cullinan 1997). These stress responses minimize the subjective interpretation of the stressor, which is useful when subjective interpretation of the stressor is considered noise variability.

In the Cold Pressor Test (CPT), participants submerge their hand in a cold water bath (0° to 6°C) for as long as can be tolerated up to a given maximum duration, typically 1 to 2 minutes (Velasco et al. 1997). The CPT reliably induces cardiovascular activation, subjective distress/discomfort, and may induce brief and modest activation of stress hormones such as adrenocorticotropic hormone (ACTH) and cortisol (McRae et al. 2006). Alcohol-dependent and non–alcohol-dependent people differ in their response to the CPT in that the former show a less robust neuroendocrine response but report more subjective distress (Brady et al. 2006). Generally speaking, the CPT does not increase craving in alcoholics, although individual differences in craving response following the CPT have been shown to predict alcohol use 1 month later (Brady et al. 2006).

Physical exercise evokes activation of the hypothalamic–pituitary–adrenal (HPA) axis, which controls the body’s major hormonal stress response, both in nonalcoholic study participants (Coiro et al. 2007; Singh et al. 1999) and alcoholics (Coiro et al. 2007). Coiro and colleagues (2007) examined how length of abstinence (4, 6, and 8 weeks) associated with stress reactivity using a stationary bicycle that measures work performed, with activity workload increasing every 3 minutes until participants reached exhaustion (approximately 15 minutes). Whereas exercise induced a significant rise in plasma ACTH and cortisol in nonalcoholics, 4-week-abstinent alcoholics failed to show an exercise-induced rise in either measure. After 6 weeks of abstinence, the endocrine response was partially normalized, and after 8 weeks of abstinence the ACTH and cortisol response was nearly identical to the nonalcoholic group (Coiro et al. 2007). Physical exercise has not, to the authors’ knowledge, been examined in a clinical laboratory setting for its ability to induce craving or drinking in alcoholics or social drinkers or to compare stress reactivity between at-risk individuals and healthy study participants.

The isometric handgrip exercise is a classic physical stressor frequently used in laboratory studies examining cardiovascular response because it reliably produces elevations in blood pressure and heart rate (Ewing et al. 1974). With this task, the participant squeezes a handgrip dynamometer as firmly as possible to determine his or her maximal handgrip strength. Then the participant is instructed to squeeze and maintain pressure at 20 to 40 percent of maximum strength for 2 to 5 minutes. No studies of the handgrip stressor alone have reported how the stressor differentially affects alcoholics versus control subjects, although studies combining the handgrip exercise with additional stressors produced a blunted cortisol response in alcoholics compared with nonalcoholics (Bernardy et al. 1996). To date, the isometric handgrip stressor has not been used as an applied stressor to examine its effect on craving or drinking.

In general, physical stressors are best suited to study specific mechanisms underlying the stress response that may be perturbed as a result of repeated alcohol exposure. In addition, they may be used to characterize individuals as high- and low-stress responders and examine subsequent response to non-physical stressors (Singh et al. 1999). Physical stressors do not mimic stressful experiences that likely lead to drinking or relapse in the real world, so if the research question is how a stressor affects subsequent alcohol use or urge to use, psychological stressors may be a better choice.

## Psychological Stressors

Psychological stressors, by definition, involve cognitive assessment of the stressor and can be classified broadly into three main categories—performance tasks, social interaction tasks, and individualized guided imagery or other mood-inducing stimuli, although a stressor may include more than one type.

### Performance Tasks

Performance tasks are designed to induce a stress response by challenging a person to solve a problem that is either difficult in its own right or is made difficult with stringent time constraints. The stress response is typically characterized by subjective measures (e.g., degree of reported distress, frustration, and anger) and objective measures, such as cardiovascular responses and electrical conductance of the skin (i.e., skin conductance, which varies with the amount of sweat produced).

The mirror star-tracing task requires participants to trace a star while being provided misleading visual feedback regarding how to adjust one’s course (e.g., up/down and left/right are reversed). Although there are no known studies examining the effects of this task specifically in alcoholics, a similar procedure has been used in individuals in a general substance abuse treatment facility. The degree of distress/frustration induced by the task (as measured by the participant discontinuing the task) was negatively related to subsequent retention in treatment (Daughters et al. 2005). Correlational in nature, these results do not yet reveal whether the mirror star-tracing task can be used to evaluate an individual’s stress reactivity or its effect on alcohol consumption or craving.

In the computerized Paced Auditory Serial Addition Task (PASAT) (Lejuez et al. 2003), numbers are sequentially presented on a computer screen and participants are requested to sum consecutive numbers in sets of two. For example, values 2 and 4 are presented (correct answer = 6) and then followed by 8 (correct answer = 12, because 8 is added to the last number presented and not the sum previously derived). The PASAT has been shown to induce changes in skin conductance, elevations in heart rate, and emotional distress (Lejuez et al. 2003) and small increases in salivary cortisol (Pratt and Davidson 2009). High PASAT-induced distress predicted early dropout from a substance abuse treatment program (Daughters et al. 2005). However, the PASAT did not induce craving or subsequent drinking in a clinical laboratory study with alcoholics (Pratt and Davidson 2009).

### Social Interaction Tasks

Performance tasks lack an important element of inducing psychological distress—the threat of social evaluation (see Dickerson and Kemeny 2004 for a review). In healthy men, a performance test increased blood pressure by 5 to 10 mmHg, whereas a social interaction task induced changes of twice that magnitude (Dimsdale et al. 1988). Not surprisingly, social-interaction tests also have been shown to induce greater cardiovascular, neuroendocrine, and subjective responses than physical stressors (Dimsdale et al. 1988; McRae et al. 2006).

A variety of methods are available to induce social interaction stress, including methods to induce feelings of social rejection and self-consciousness about physical appearance (Sayette et al. 2001; Stroud et al. 2000), but the gold standard of social interaction stressors is the Trier Social Stress Test (TSST). The TSST (Kirschbaum et al. 1993) is a widely used standardized social stress procedure in which the participant is sequentially exposed to three unique stress-inducing situations: a preparation phase, an interview phase, and a mental arithmetic phase. In the preparation phase, the participant is instructed to prepare his or her talking points for a subsequent mock job interview. A few minutes later, the participant engages in a mock job interview, presenting to confederates who are trained to remain stoic during the interview process. Finally, the participant performs a serial subtraction task to the audience, and if an incorrect value is given, the participant must begin again with the initial number. Each element of the TSST typically lasts 5 minutes, for a total exposure time of about 15 minutes (Kirschbaum et al. 1993).

The TSST has been shown to evoke a robust and predictable response curve for subjective distress, heart rate, blood pressure, cortisol, and ACTH (Kirschbaum et al. 1993; Singh et al. 1999). Generally speaking, the TSST induces a two- to fourfold increase in cortisol levels (Kirschbaum et al. 1993; Singh et al. 1999). Because the TSST yields such a marked and objectively measurable stress response, it is especially well suited for studies in which stress reactivity outcomes are of particular interest.

The TSST has been widely used to compare the magnitude of stress reactivity and stress-response dampening by alcohol in individuals at risk for alcoholism, as defined by heavy drinking or a family history of alcoholism. These studies generally support that at-risk individuals differ from healthy counterparts on both stress reactivity and stress-response dampening (Croissant and Olbrich 2004; Uhart et al. 2006; Zimmermann et al. 2009). Research also generally suggests that alcoholics and nonalcoholics differ in their response to the TSST (Lovallo et al. 2000; McRae et al. 2006; Munro et al. 2005).

Relatively few studies have examined the effect of the TSST or other social interaction–based stressors on alcohol craving or consumption. The TSST has been shown both to induce craving (Nesic and Duka 2008) and also to have no effect on craving (de Wit et al. 2003; Nesic and Duka 2006) in social drinkers. In studies of stress-induced craving or drinking in problem drinkers, Thomas and colleagues (2011*a*) found that the TSST increased drinking but not alcohol craving or alcohol cue reactivity (Thomas et al. 2011*a*,*b*) in non–treatment-seeking alcoholics.

In general, the TSST is especially well suited for research questions related to stress reactivity—for example, variables that predict stress reactivity, such as family history of alcoholism (Uhart et al. 2006) and the effect of alcohol on the stress response (Zimmermann et al. 2009). The TSST may be valuable for examining stress-induced drinking in a laboratory setting (Thomas et al. 2011*a*), but more studies are needed to replicate this finding. Most clinical laboratory studies conducted to examine whether stress induces drinking or craving have relied on personalized (rather than standardized) stressors, such as individualized guided imagery.

### Individualized Guided Imagery

Significant individual differences exist in what is interpreted as stressful. Guided imagery paradigms use stimuli that are individually calibrated for emotionality and stressfulness to induce emotion and stress reactivity while approximating real-life situations (for review, see Sinha 2009). The individualized guided imagery procedure involves developing personalized imagery scripts for both stressful and nonstressful situations. Scripts are developed based on the participants’ own descriptions of each situation. Individualized scripts are then recorded on an audiotape and presented to the participant in the laboratory with instructions to imagine the situation “as if it were happening right now,” so that the relevant mood can be induced. Researchers then compare responses to stressful and nonstressful scripts, as well as their respective effects on substance use variables of interest (e.g., craving).

Individualized stress imagery has been shown to increase negative emotions, and to a lesser degree, cardiovascular activity, ACTH, and cortisol (Sinha 2007). The procedure has been used to identify differences in stress responses between social drinkers and people who are alcohol dependent (Sinha et al. 2009) and to show that alcohol and drug craving is elevated following exposure to stressful versus neutral imagery cues in individuals with alcohol dependence (Cooney et al. 1997; Fox et al. 2007; Sinha 2007). It is unknown whether guided imagery stressors increase drinking in alcoholics, although it has been shown that severity of craving following exposure to stressful scripts predicted time to relapse following inpatient treatment (Sinha et al. 2011).

Although guided imagery is the most widely used technique in alcohol and addiction research to induce a specific mood, other mood induction approaches include exposure to somber or otherwise emotionally laden music (Birch et al. 2004; Grant et al. 2007; Jansma et al. 2000; Willner et al. 1998) or to sad or disturbing images (Mason et al. 2008). In general, these techniques are effective in inducing the target mood, although amenable to confirmation only with subjective indices. Only negative mood induction using music has been shown to induce the urge to drink and only in certain subgroups, such as those who report using alcohol as a coping strategy (see Birch et al. 2004; Grant et al. 2007).

Psychological stressors have the advantage of modeling stressors, or at least stress-induced emotions (anxiety, dread, frustration, and embarrassment), that individuals encounter in the real world. If psychological stressors are used and objective confirmation of the stressor is not feasible, investigators are encouraged to use subjective measures that capture a range of emotions, where the participant can report changes in fear, anger, frustration, humiliation, etc., and not simply the level of “stress” experienced. Visual analog scales querying multiple emotions (see de Wit et al. 2003) and standardized instruments (see sidebar) allow the respondent to more fully describe his or her subjective interpretation of the stress experience.

## Pharmacologic Stressors

The primary events of the stress response are the release of corticotropin-releasing factor (CRF) and vasopressin from the hypothalamus, resulting in the release of ACTH from the pituitary gland to stimulate the adrenal cortex to release cortisol. Cortisol then inhibits the release of CRF and ACTH in a negative-feedback loop. Pharmacological stressors have been used primarily to identify specific disruptions in this system that occur as a result of alcohol dependence or pre-existing differences between at-risk and low-risk individuals.

CRF, ACTH, and cortisol release can be induced through a number of different agents, including glucose-depriving medications such as insulin (Costa et al. 1996) or 2-deoxyglucose, nicotine (Matta et al. 1998), and alcohol itself. Agents that mimic the actions of serotonin (i.e., serotonergic agonists), such as fenfluramine (Anthenelli et al. 2001), meta-chlorophenylpiperazine (mCPP) (Krystal et al. 1996), and citalopram (Mondelli et al. 2006) also increase hypothalamic CRF, although direct pituitary and adrenal effects also have been posited (Dinan 1996). In addition, agents that block opiate receptors (i.e., antagonists, such as naloxone) block opioid tonic inhibitory modulation of CRF and so result in release of ACTH and cortisol (Inder et al. 1995). Another approach is to apply synthetic or species-specific versions of CRF and ACTH. The administration of ovine CRF (oCRF) mimics the effect of naturally occurring CRF on the pituitary, and a synthetic derivative of ACTH (i.e., cosyntropin) directly stimulates cortisol release from the adrenal cortex. In addition, researchers have used synthetic steroid hormones (i.e., glucocorticoids, such as dexamethasone) to examine the integrity of negative-feedback mechanisms (Khan et al 1984).

Most pharmacological stressors have been used to examine how people with alcohol dependence or risk of developing dependence via positive family history differ from nonaffected study participants or how time in recovery affects the HPA axis. For example, regarding family history, administration of alcohol as a pharmacologic stressor resulted in a blunted cortisol response in young men with an alcohol-dependent biological father but not in a comparison group (Schuckit et al. 1987). On the other hand, opiate receptor antagonists have resulted in higher ACTH and/or cortisol response in people with a positive family history of alcoholism compared with those with a negative family history (King et al. 2003; Wand et al. 2001). Neither oCRF (Waltman et al. 1994) nor cosyntropin (Wand et al. 1999) showed differences between family history positive and negative individuals.

Results from pharmacological challenge studies with people who are alcohol dependent tend to be more consistent. Alcoholics typically exhibit a muted ACTH or cortisol response to these stressors, including insulin (Costa et al. 1996), nicotine (Coiro and Vescovi 1999), naloxone (Inder et al. 1995), and mCPP (Krystal et al. 1996). Some notable exceptions include exaggerated cortisol responses to fenfluramine (Anthenelli et al. 2001) and 2-deoxyglucose (George et al. 1994). Both oCRF and cosyntropin produce lower ACTH and/or cortisol responses in alcohol-dependent men compared with nonalcoholic men (Adinoff et al. 1990, 2005; Inder et al. 1995; Wand and Dobs 1991).

**Quantifying Stress Reactivity**

In stress-induction studies, stress response optimally is measured with both subjective and objective indices because together these provide the strongest evidence for the internal validity of the stressor. Data from objective and subjective measures also may provide complementary (not necessarily overlapping) information. For example, a robust stress-induced change in cortisol is not necessarily correlated with a more intense experience of distress (Dickerson and Kemeny 2004). Both objective and subjective outcomes should be assessed prior to, and multiple times following, the stress-induction procedure to reveal the time course of the stress response. The following sections describe the subjective and objective indices most commonly used in stress-induction studies to confirm and quantify stress reactivity in alcohol research (for a more comprehensive review of assessments, see Davis et al. 2007).

**Subjective Measures of Stress Reactivity**

Subjective measures of stress reactivity quantify the individual’s experience of distress or discomfort via his/her self-report. The most commonly used subjective measure of stress reactivity is a 7- (1 to 7) to 11- (0 to 10) point Likert scale or a visual analog scale (VAS) (measured along a 100-mm line) on which the respondent rates his/her severity of distress. For Likert scale items, the low and high values may reflect level of agreement with a statement such as “I feel stressed” or may reflect the degree of a stressed state (“none at all” to “extreme”). For VAS items, the line is labeled “none at all” at the left end and “the most I’ve ever experienced” at the right end, and the respondent indicates his/her current state by placing a mark along the line. The location of the mark is measured in millimeters from the end with the low-severity anchor. For both the Likert and VAS scale question, the state assessed may be a single concept, such as “distress” or “stress,” or several terms may be used with each rated singly, such as fear, nervousness, anger, or anxiety. Likert and VAS scales also have been used to index feelings that are in contrast to the experience of distress, such as neutral, happy, pleasant, relaxed, and calm, with the rationale that such feelings should decrease as aversive states are induced by the stressor. Results from each descriptor typically are analyzed separately rather than summed to compute a total score. In addition to measuring what they intend to measure (i.e., having face validity), these scales have been psychometrically evaluated and have been shown to adequately capture current feelings of anxiety (Davey et al. 2007). They also are simple and inexpensive to administer, and collecting results does not require extensive time. As a result, nearly every stress-induction study includes at least one self-reported Likert scale or VAS item to quantify participants’ distress.

Standardized questionnaires also are used to assess distress in stress-induction studies. These instruments include multiple items that are used to compute a total score and/or subscale scores. Standardized instruments also allow comparison of results across studies. Four commonly used instruments in stress induction challenges in alcohol research are the State–Trait Anxiety Inventory (STAI; 20 items) (Spielberger 1983), the Positive and Negative Affect Schedule (PANAS; 20 items) (Watson et al. 1988), the Profile of Mood States (POMS; 65 items) (McNair et al. 1971), and Izard’s Differential Emotions Scale (DES; 30 items). Each of these instruments has sound psychometric properties, as reported in their source references (see Boyle 1984 for the DES). Because of the length of these instruments, they may not be suitable for repeated assessment over a short time frame and may induce participant fatigue. To minimize these problems, investigators often use shorter versions such as the 6-item STAI (Marteau and Bekker 1992) or administer selected subscales from the instruments, such as the tension–anxiety subscale (9 items) from the POMS.

**Objective Measures of Stress Reactivity**

Objective measures of stress reactivity quantify physiological changes that reflect activation of the hypothalamic–pituitary–adrenal (HPA) axis and the sympathetic nervous system. These include neuroendocrine measures, such as levels of the stress hormones adrenocorticotropic hormone (ACTH) and cortisol, and physiologic measures such as heart rate, blood pressure, and, less commonly, skin conductance. Neuroimaging, which recently has been adopted as an additional objective assessment of stress reactivity, can be used to show activation of brain areas associated with regulating emotion (see Sinha and Li 2007). As the latter is restricted in its use to specific stressors amenable to delivery in the scanner, the following section focuses on the objective assessments of stress reactivity that may be collected following any stress induction procedure.

ACTH and cortisol are the two neuroendocrine measures most often used to index stress reactivity and specifically HPA axis activation. ACTH is produced and secreted by the anterior pituitary gland to promote the adrenal cortex to release cortisol. ACTH must be measured from blood, whereas cortisol may be measured in either blood or saliva. Salivary cortisol reflects the binding protein-free fraction and thus the biologically active form of cortisol and may be less susceptible to interference by oral contraceptives (Vining et al. 1983). Although either serum or salivary cortisol can index HPA axis activity, salivary cortisol provides a more accurate depiction of active circulating cortisol (Gozansky et al. 2005).

If blood is collected in the challenge, care must be taken not to induce “noise” stress by repeatedly sticking the participant to draw the sample. Thus, a peripheral venous catheter is recommended. Timing the collection of samples also is relevant, particularly for cortisol, because there is robust diurnal variation in cortisol levels. Investigators can establish a model of baseline levels of cortisol by collecting samples several times prior to the stress manipulation.

The expense of collecting and measuring ACTH and cortisol may be prohibitive for some studies, and investigators may therefore use cardiovascular activity such as heart rate and blood pressure to objectively assess stress reactivity. These measures can be collected with automated equipment, so no extensive training is needed. Heart rate is assessed by beats per minute; systolic and diastolic blood pressure is measured in millimeters of mercury (mmHg). Mean arterial pressure, which reflects the average arterial pressure over a complete cycle of one heartbeat, is computed using systolic and diastolic pressure values. It is especially well-suited for stress-induction procedures because it indexes the role of the sympathetic and parasympathetic systems in regulating blood pressure. Heart rate variability, specifically respiratory sinus arrhythmia, can be calculated from the heart rate as a noninvasive index of parasympathetic control of cardiac activity (Bernstein et al. 1993).

In summary, confirming the validity of the stress-induction procedure is critical to evaluating the effects of the stressor (or lack thereof) on alcohol-related outcomes such as craving or consumption, or how at-risk and alcohol dependent people differ from others in response to an applied stressor. Depending on the specific research question, the stressor selected, and logistical constraints, investigators may select certain indices over others. Given the host of subjective and objective measures of stress reactivity available, however, investigators should seek to quantify the stress response of participants with both subjective and objective data.

ReferencesBernsteinGGCacioppoJTQuigleyKSRespiratory sinus arrhythmia: Autonomic origins, physiological mechanisms, and psychophysiologic implicationsPsychophysiology301831961993843408110.1111/j.1469-8986.1993.tb01731.xBoyleGJReliability and validity of Izard’s differential emotions scalePersonality and Individual Differences57477501984DavisGLAl’AbsiMHovlandJAssessment of stress in research and clinical settingsAl’AbsiMStress and Addiction: Biological and Psychological MechanismsBurlington, MAElsevier2007265284DickersonSSKemenyMEAcute stressors and cortisol responses: A theoretical integration and synthesis of laboratory researchPsychological Bulletin130335539120041512292410.1037/0033-2909.130.3.355GozanskyWSLynnJSLaudenslagerMLKohrtWMSalivary cortisol determined by enzyme immunoassay is preferable to serum cortisol for assessment of dynamic hypothalamic-pituitary-adrenal axis activityClinical Endocrinology6333634120051611782310.1111/j.1365-2265.2005.02349.xMcNairDLoorMDropplemanLProfile of Mood StatesSan Diego, CAEducational and Industrial Testing Service1971MarteauTMBekkerHThe development of a six-item short-form of the state scale of the Spielberger State-Trait Anxiety Inventory (STAI)British Journal of Clinical Psychology313013061992139315910.1111/j.2044-8260.1992.tb00997.xSinhaRLiCSImaging stress- and cue-induced drug and alcohol craving: Association with relapse and clinical implicationsDrug and Alcohol Reviews262531200710.1080/0959523060103696017364833SpielbergerCDManual for the State-Trait Anxiety Inventory (Form Y)Palo Alto, CAConsulting Psychologists Press1983ViningRFMcGinleyRAMaksvytisJJHoKYSalivary cortisol: A better measure of adrenal cortical function than serum cortisolAnnals of Clinical Biochemistry203293351983631683110.1177/000456328302000601WatsonDClarkLTellegenADevelopment and validation of brief measures of positive and negative affect: The PANAS scalesJournal of Personality and Social Psychology54106310701988339786510.1037//0022-3514.54.6.1063

Although pharmacological stressors have not historically been used to examine the effects of stress on subsequent craving or drinking, these stressors more recently have been applied to study whether they can induce alcohol craving in alcoholics (Umhau et al. 2011). The α-2 adrenergic antagonist, yohimbine, induces anxiety (Holmberg and Gershon 1961) yet has inconsistent evidence of inducing craving (Krystal et al. 1994; Umhau et al. 2011). The stressor mCPP has been more effective at inducing not only robust subjective distress but also enhanced alcohol craving (George et al. 1997; Krystal et al. 1994; Umhau et al. 2011). Because pharmacological stressors have the advantage of being directly applicable to preclinical models, and vice versa, they are especially relevant for translational research efforts.

## Summary

The relationship between stress and alcohol use is complex, and clinical laboratory studies in which an acute stressor is applied allow researchers to further clarify links between stress reactivity and alcohol use and abuse through systematic study. Research has identified the architecture of the stress response, and evidence across classes of stressors—physical, psychological, and pharmacologic—generally supports the hypothesis that both people with alcohol dependence and those at risk for alcoholism (e.g., heavy drinkers or those with positive family history of alcoholism) differ from comparison groups in their response to applied stressors. Whether this difference contributes to the development of alcohol problems or is simply a phenotypic marker of pre-existing risk is yet unknown. How stress results in alcohol seeking, craving, and/or relapse in individuals with alcohol dependence also is not well understood, but because it has important treatment implications, it is a fruitful area for future study. For example, clinical laboratory studies in which stressors are applied can result in clinical models in which investigators can study whether promising treatments diminish the ability of stress to enhance motivation to drink and whether such treatments may alter stress reactivity (Kosten 2011).

The stressors described in this article frequently are used in clinical laboratory settings and have empirical support for their ability to induce a measurable stress response. Ideally, the ability of an applied stressor to induce stress (i.e., internal validity) is confirmed by both objective and subjective indices. The optimal stress-induction procedure is determined by the specific research question. For example, guided imagery stressors induce subjective distress as well as alcohol craving but may not induce robust changes in stress reactivity as indexed by objective measures. Conversely, the TSST is considered the gold standard for eliciting neuroendocrine reactivity (Dickerson and Kemeny 2004) but has shown inconsistent effects on inducing the urge to drink. If the research question involves understanding what part of the HPA axis cascade is perturbed, pharmacological stressors may be optimal; they also present an exciting opportunity in translational research studies. If the research question is to examine differences between groups on stress reactivity, a stressor that affords both objective and subjective confirmation is recommended. If the study seeks to determine what type of person is likely to be provoked to craving or alcohol consumption by stress, psychological stressors that approximate real-life situations (such as guided imagery and possibly the TSST) may be the best choice.

The complexity of the relationship between stress and alcohol use has resulted in an empirical base with more questions than answers. Research does show that stress is undoubtedly related to alcohol use and vice versa (Cooney et al. 1997; Sinha 2007). Clinical laboratory studies that examine the effects of acute stressors on alcohol-relevant outcomes are critical to elucidating this complex relationship because they provide the opportunity to determine mechanistic links between stress reactivity and alcohol use and abuse, thus providing direction for optimal treatment and prevention efforts.
